# Communicating under medical patriarchy: gendered doctor-patient communication between female patients with overactive bladder and male urologists in Hong Kong

**DOI:** 10.1186/s12905-015-0203-4

**Published:** 2015-05-29

**Authors:** Judy Yuen-man Siu

**Affiliations:** David C. Lam Institute for East-West Studies (Environment, Health, and Sustainability working group), Hong Kong Baptist University, Hong Kong, China

**Keywords:** Female patients, Male urologists, Communication, Overactive bladder, Hong Kong

## Abstract

**Background:**

Gender differences between patients and doctors markedly influence the quality of communication in treatment processes. Previous studies have shown that communication between patients and doctors of the same gender is usually more satisfactory, particularly for female patients. However, in Hong Kong, where urology is a male-dominated specialty, female patients typically require medical care from male doctors for diseases such as overactive bladder (OAB). The literature about gender-related doctor-patient communication predominantly involves people in non-Chinese communities, with few studies conducted with Chinese populations. However, the differences between Western and Chinese cultures are expected to result in different treatment and communication experiences. Furthermore, OAB has received little attention in many Chinese communities; few studies in the literature address the communication quality between OAB patients and their urologists in Chinese communities, particularly regarding female OAB patients’ experiences when seeking treatment from male urologists. This study, therefore, investigated the doctor-patient communication between female OAB patients and male urologists in Hong Kong.

**Methods:**

This study adopted a qualitative research approach by conducting semistructured interviews with 30 female OAB patients on an individual basis from April 2012 to July 2012. The participants were purposively sampled from a patient self-help group for OAB patients in Hong Kong.

**Results:**

The participants’ communication experiences with male urologists were unpleasant. Embarrassment, feelings of not being treated seriously, not being understood, and not being given the autonomy to choose treatment approaches prevailed among the participants. Furthermore, the perceived lack of empathy from their urologists made the participants’ communication experiences unpleasant.

**Conclusions:**

The gender and power differential between the participants and their urologists, which was contributed by the social and cultural values of patriarchy and doctors’ dominance in Hong Kong, made the participants’ communication with the urologists unpleasant and difficult. Poor doctor-patient communication can endanger patients’ treatment compliance and thus the treatment outcome. Although altering such social and cultural values would be difficult, providing complementary chronic care services, such as nurse-led clinics as well as support and sharing from patient self-help groups, might be a possible solution.

## Background

Gender is a crucial factor influencing interpersonal communication. Gender differences between patients and their doctors markedly influence the quality of communication in treatment processes [1, 2], thus ultimately affecting the quality of treatment experienced by patients as well as their treatment compliance and outcomes [1]. A positive treatment experience can enhance treatment compliance and thus the treatment outcome, whereas a negative treatment experience has the opposite effect [3].

A previous study showed that patients are often satisfied with their treatment when female and male doctors exhibit distinct nonverbal behaviours [2], and they are more satisfied with doctors who conform to gender roles [2]. Patients and doctors of the same gender typically have more satisfactory communication in treatment processes [4], particularly regarding female patients [4]. Moreover, when patients have conditions that they perceive to be sensitive or embarrassing, they tend to communicate better with doctors of the same gender [4, 5].

However in some diseases, female patients unavoidably require medical care from male doctors. This is particularly the case for female patients with overactive bladder (OAB) in Hong Kong, where urology is a male-dominated specialty [6]. OAB is a chronic bladder dysfunction with high prevalence worldwide. In some population-based studies conducted in Europe and in Canada, it was estimated that the overall prevalence of OAB in individuals aged over 40 was 16.6 % [7] and 13.9 % [8] respectively. In Hong Kong, the Hong Kong Urological Association reported that the number of OAB cases has increased; a survey conducted in 2009 revealed that approximately 15 % of the sampled 1300 respondents aged over 40 had conditions diagnosed as OAB [9]. Patients with OAB have poorer quality of life than that of patients suffering from other kinds of incontinence [10]. OAB primarily affects women, for whom the symptoms are more bothersome [11]. Typical symptoms of OAB include “urinary urgency, usually accompanied by frequency and nocturia, with or without urgency urinary incontinence, in the absence of urinary tract infection or other obvious pathology” [12]. Treatment and management of OAB include lifestyle modification, behavioural therapy, pharmacotherapy, neuromodulation, botulinum toxin therapy, and surgical intervention [13].

In Hong Kong, approximately 15 % of the population had OAB in 2009 [14], and the prevalence is presumably rising because of the increase in education coverage, which has raised public awareness about this chronic bladder condition. OAB induces substantial and multidimensional impacts on patients, and additional lower urinary tract symptoms have an even greater impact [15]. Because of its symptoms, patients often perceive OAB to be an embarrassing disease [16]. Feeling embarrassed with a condition is one of the most common factors for patients to delay seeking treatment [17, 18]. Even for patients who receive treatment for OAB, communicating about the disease with their doctors can be difficult [18, 19]. This affects their treatment experiences and thus the treatment outcomes.

### Significance

In Hong Kong, patients with OAB are clinically managed by urologists. In 2014, 112 urologists registered with the Medical Council of Hong Kong, approximately 94 % of whom were male [6]. Although most female patients seeking treatment are unconcerned about the gender of their doctor [20, 21], other studies have reported the opposite, particularly in which patients were from non-Western countries or had diseases that they perceived to be sensitive [22–24]. However, the literature on gender-related doctor-patient communication predominantly involves non-Chinese communities. The differences between Western and Chinese cultures are expected to result in different treatment and communication experiences. Therefore, this study was conducted to fill this gap in the literature. Furthermore, OAB has received little attention in many Chinese communities; few studies in the literature address the communication quality and problems between OAB patients and their urologists in both Western and Chinese communities, particularly regarding female OAB patients’ communication experiences when seeking treatment from male urologists. This study, therefore, investigated the experiences of female patients with OAB communicating with male urologists in Hong Kong. This study was conducted to address the following research questions:

1. What did the participants experience when communicating with male urologists?
2. How did the participants perceive the quality of communication with the male urologists?
3. What communication difficulties did the participants encounter when receiving treatment from male urologists?
4. How did the participants’ experiences in communicating with male urologists affect their treatment experience and their motivation to receive treatment?
5. How did Chinese cultural values about gender and the hierarchical difference between doctors and patients combine to affect the participants’ experiences in communicating with male urologists?

This study was inspired by my earlier research on OAB patients, covering the illness experiences of female patients – in which all of them reported having encountered unpleasant treatment experiences [18] – as well as the doctor shopping behaviour of these patients [25]. These earlier articles showed that the patients’ doctor shopping behaviour was to be induced by unpleasant illness experiences [25], and one of the most noteworthy reasons for their unpleasant treatment experience was the difference in gender [18]. However, in these earlier articles, my main aim was to examine the patients’ general illness experiences in encountering OAB [18] as well as what had caused their doctor shopping behaviour [25]. Communication experiences and difficulties that were related to and caused by the gender difference between doctors and patients, however, were not the research questions of these earlier studies. Being treated by a urologist of the opposite sex made it difficult for the patients to communicate effectively with their doctor. My research and training background in medical anthropology without clinical medicine training has thus attracted my interest in the social and cultural factors embedded in doctor-patient communication. Therefore in this article, I investigate further about the communication experiences and feelings of female OAB patients in Hong Kong, who are not well recognised and receive little attention and support. To achieve this, I conducted another set of semistructured interviews individually with the patients for this article, investigating their communication experiences with their doctors in opposite sex under the influence of gender in particular.

## Methods

To elucidate the experiences of female OAB patients in communicating with male urologists, this study adopted a qualitative approach of data collection with individual semistructured interviews and a phenomenological approach of data analysis.

### Ethical considerations

Ethical approval on the study was obtained from the Committee on the Use of Human and Animal Subjects in Teaching and Research at Hong Kong Baptist University prior to conducting this study. Participation in this study was voluntary. Prior to the interviews, all participants were provided an information sheet explaining the purpose and nature of the study. For the sake of clarity, the information sheets were written in traditional Chinese, which is the participants’ native language. Where necessary, further explanation and clarification were provided verbally. Finally, signed informed consent was obtained from each participant, and they were informed about the use of data in academic publications, and of their rights and freedom to withdraw from the study.

To ensure the participants’ privacy, no identifying details were recorded in audio or in the coded data. All participants were designated with codes in the interview transcripts. The data were stored in locked cabinet and treated with strict confidentiality, and the data were not shared with any third party. All audio recordings of the interviews were destroyed after transcribing them.

### Data collection

In this study, data were collected by adopting a qualitative data collection approach involving in-depth individual semistructured interviews. Individual interviews were conducted instead of focus group interviews to facilitate a more in-depth collection of data, and it is particularly suitable for the investigation of sensitive topics [26]. Thirty participants were purposively sampled at a patient self-help group for OAB patients in Hong Kong from March to April 2012. The patient self-help group was established in 2008 [27]. At the time of this study, the group had nearly 300 members, approximately 200 of whom were females. The participants were purposively sampled based on the following inclusion criteria: (a) being a 21–59-y-old woman, (b) having a confirmed diagnosis of OAB by a medical practitioner, (c) having received treatment from a male urologist, and (d) being of Hong Kong Chinese ethnicity. The 30 women were purposively sampled for this study because of their treatment experiences with male urologists. Those diagnosed with other types of urinary incontinence (including stress incontinence, overflow incontinence, mixed incontinence, structural incontinence, and functional incontinence), and those who had not yet received a confirmed OAB diagnosis prior to the sampling period were excluded from this study.

Prior to the interviews, a new set of interview question guide was developed based on the data of my earlier studies on the illness experiences of OAB patients [18] and their doctor shopping behaviour [25], as well as on the past literature about doctor-patient communication. The interview question guide was used throughout the interviews to ensure that the interviews were focussed on the research questions [26]. To ensure that the questions would be understood and that the interviews would proceed smoothly, the interview question guide was tested on five women who were a similar age and had a similar educational background as the participants. These interview questions were open-ended, aiming at investigating the participants’ experiences and difficulties when communicating with their urologists during treatment, as well as their feelings and coping strategies. The participants had a high degree of flexibility in expressing their views, feelings, and experiences [28]. Responding to each participant, additional follow-up questions were asked in order to probe for further information from the participants. The questions in the interview question guide were specially developed for this study, included the followings:

1. What gender do you prefer your doctor to be when you are seeking medical advice?
2. What is the gender of your usual doctor(s)?
3. Does your preference of doctor’s gender differ by disease? If yes, how and why does it differ?
4. How do you feel when encountering a male doctor regarding common diseases or conditions?
5. How do you feel when consulting a male doctor about your bladder condition?
6. How comfortable are you when communicating with male doctors during consultation for your bladder condition?
7. Do you think there is any difference in your communication when consulting a male doctor or a female doctor about your bladder condition? If yes, can you explain the difference? Why is there such a difference?
8. How do you cope with your feelings/emotions when communicating with a male doctor regarding your bladder condition?
9. From your experience and observation, how comfortable are the male doctors when communicating with you regarding your bladder condition?
10. What do you think about how doctors communicate with you regarding your bladder condition? Does this influence your feelings and treatment experience? If yes, how?

The in-depth semistructured interviews were conducted on an individual basis from April to July 2012. All interviews were conducted by the researcher to ensure quality and consistency. This approach reduced the likelihood of collecting insufficient data and the risk of having data flaws from introducing another interviewer. Moreover, how participants perceive an interviewer can influence how they interact during an interview [29]. As a researcher in medical anthropology with no clinical medicine training, my role as a non-doctor interviewer with the participants was more equal, which reduced the participants’ sensitivity and their sense of inferiority regarding the expert power of doctors as well as the hierarchical difference between doctors and patients in Chinese communities. This reduced the risk of potentially inaccurate reports by the participants. All interviews were conducted in Cantonese Chinese—the mother tongue of the participants and the researcher—so that the participants could express their views and experiences freely without any language barrier [28].

The interviews were conducted privately in a classroom at Hong Kong Baptist University. The classroom was located next to female washroom for the participants’ convenience. Each interview lasted from 1 h and 45 min to 2 h, and was audio-recorded with their consent. Because of their chronic bladder condition, the interviews were paused according to the participants’ requests. To compensate them for their time, each participant was given a supermarket cash coupon of HK$100 (approximately €12) on completion of the interviews.

### Data analysis

Data saturation was achieved. A phenomenological approach was adopted to examine the experiences of the participants and how they described these experiences [30]. Interviews were transcribed verbatim and then translated into English. Back-translation was performed to ensure the translated transcripts did not distort the original meanings of the participants. Subsequently, the interview transcripts were segmented into meaning units, collapsed into categories, and eventually organised into themes through the process of abstraction and constant comparison. Repetitive codes and themes were noted and highlighted, and new thematic codes that emerged from the data were added to the coding list. Coding schemes were developed [26] according to an inductive coding process by allowing the discovery of patterns of behaviours and thoughts [28]. A coding table identifying the themes, categories, and codes with supporting interview quotes was constructed. Memos were used to record ideas and commentary during the interview and coding process. A codebook was maintained to record special data [28]. The analytic procedures, codings, and findings were documented in the codebook to assure consistency and accuracy of the data. Because the data collection and analysis of this study were conducted by a single researcher, a recoding process was conducted 1 month after the first coding as a cross-analysis to eliminate possible subjectivity and bias, and to enhance the validity and reliability of the coded data.

### Research rigor

Credibility for the study was established by performing validity checks with the participants. The participants were invited to check the transcribed interviews to ensure that the transcription process did not distort their intended meanings. Direct interview quotations from the participants were included in my analysis to ensure that their ideas were clearly represented. Neutrality was established, and the findings were grounded in the interview data and not in researcher’s bias, motivation, or interest. Reliability and confirmability were established through coding and recoding of the transcripts to ensure that the codings and categories were clear and free of ambiguity and overlaps.

## Results

### Research participants

All 30 participants were Hong Kong Chinese women, and were aged between 28 and 55 y at the time of study. Most participants were in the age group of 30s to 50s. The sampled women comprised 24 full-time working professionals and 6 homemakers. The 24 working participants were employed in various sectors, including civil service, education, social welfare, commerce and finance, sales and retailing, information technology, and administrative and clerical sectors. Four of the homemakers had a part-time job, working for no more than 30 h per week.

The length of time since diagnosis was 1–6 y at the time of this study, although participants had symptoms of urinary frequency, urgency, and incontinence for 5–11 y. None of the participants received diagnosis of OAB immediately after symptoms presented. They had suffered from the symptoms for at least 2 y before their diagnosis of OAB, and some of the participants had suffered for 5 y before obtaining a diagnosis. Prior to OAB diagnosis, the participants mostly attended primary care general practitioners for treatment, and bladder infection was a common diagnosis. They received a diagnosis of OAB only on referral to a urologist for further assessment.

All participants continued receiving regular follow-up treatment from male urologists, and were under the clinical care of them for at least 2 y at the time of this study. Most participants received follow-up treatment in urology clinics at public hospitals. Participants who underwent followed-up treatment at public hospitals were not allowed to choose their doctor; instead, they were assigned to see male urologists for most of the follow-up sessions. Although a few participants received follow-up treatment from private practice urologists and were able to choose the doctors they preferred, they commented difficulty in choosing a female urologist. During follow-up treatment, the participants were required to undergo urinalysis, receive a physical examination, have their bladder scanned, undergo urodynamic study, receive bladder training, and adjust their oral medication. They were required to maintain a voiding diary under supervision. For some patients, bladder instillation therapy was also performed during follow-up.

### Themes

Because of the gender difference, the participants regarded their communication with their urologists in the treatment as unpleasant. During their communication, embarrassment, feelings of not being treated seriously, not being understood, and not being given autonomy to select treatment approaches prevailed among the participants. Furthermore, the participants commonly perceived their urologists to lack empathy towards their suffering. Although a few unpleasant experiences were more due to the structural interaction between doctors and patients, gender differences between the participants and their urologists remained the most prominent explanation for the participants’ unpleasant experiences in communicating with their urologists.

#### Embarrassment

The symptoms of OAB are embarrassing for affected patients, and this was particularly the case for the participants. Because of the gender difference, all of the participants felt embarrassed when communicating about the symptoms to their urologists, who were men, as indicated by the following participant’s comment:

I wish I could choose to see a female doctor. Seeing a female doctor is much less embarrassing; but you know, I cannot choose doctors in public hospitals. Even for a common cold or flu, I would see a female doctor; so it is really embarrassing for me to see a male doctor for my bladder; you know the location of bladder… [the participant’s face turned red]. It is even more uncomfortable and embarrassing for me to talk about my symptoms to a male doctor. [P2]

In many cases, the participants felt embarrassed during diagnostic procedures, physical examinations, and tests related to their bladder condition. The participants felt even more embarrassed because the urologists whom they were consulting were men, as indicated by the following participant’s recollection of her experience:

I feel embarrassed when male doctors perform a physical examination on me. I could still remember how embarrassing it was when the doctor did a bladder endoscopy [cystoscopy] on me, because the doctor was a man. It was just like a gynaecological check-up conducted by a male doctor. The voiding test [urodynamic study] was embarrassing, too. Going to the toilet is a very private matter for a woman; but in the test, it just made me feel that such a private matter was under surveillance by a man and made public. [P7]

The perception of bladder problems among the participants also contributed to their embarrassment. A few participants related their bladder conditions to sexual and reproductive organs. Such perceptions made the participants feel even more embarrassed when communicating with their urologists, particularly when the communication involved the participants’ sex life, as the following participant indicated:

The two organs [urinary organs and reproductive organs] are very close to each other. When a doctor examines the urinary part, he unavoidably sees the genital area. This is really embarrassing for me. Also, some doctors ask me about my sex life. I do not know whether my bladder problem is related to my sex life, but I felt very embarrassed because I was not prepared for the doctors to ask me about such a private thing. [P19]

In many cases, the feelings of embarrassment among the participants hindered them from communicating honestly and effectively with their urologists. One of the participants described how her feelings of embarrassment made it difficult for her urologist to understand her situation:

I find it very embarrassing for me to tell doctors about my bladder problems, because they are men, especially because they often talk about sensitive issues during the follow-up. When the doctor asked me some sensitive questions, I was so embarrassed that I could only sit in front of him without saying a word. I would avoid telling the doctor about the severity of my incontinence, because I think this should not affect his diagnosis and the drugs he would prescribe. I would just tell him that I need to go to the toilet often and urgently; this symptom sounds less embarrassing. Telling a male doctor about wetting trousers is very embarrassing for a woman. [P5]

#### Feelings of not being treated seriously

Most of the participants felt that their urologists tended to simplify their chronic bladder conditions and suffering given the fact that they were middle-aged women, although the participants perceived their suffering and symptoms to be bothersome and disruptive to their physical and emotional conditions. In many cases, the urologists’ responses made them feel that their condition and suffering were not being treated seriously. One of the participants shared her follow-up experience:

Doctors in public hospitals tend to simplify your problems, “from a big matter to a small matter, and from a small matter to a negligible matter” [a colloquial expression in Cantonese]. This is how I have experienced these years of treatment. Every time I mention my condition to doctors, they just say “There is nothing serious. It is not uncommon for a middle-aged woman like you.” Then I am asked to leave. Maybe they have seen such cases so many times in a day that they think that my problem is not a big deal at all. However, the suffering bothers me. I hope they can help me, but I do not feel that they are serious about my treatment. [P25]

The brevity of the consultations frequently left the participants feeling that they did not have sufficient time to communicate with their urologists during follow-up. Failing to have time to receive updates and clarification about their bladder condition was a common experience for the participants, making them feel that they were not treated seriously, as the following participant shared:

I can just see the doctor for around two to three minutes at most in each follow-up session. There is not much I can say in this amount of time. I can only talk very briefly about the updates of my symptoms, and the doctor just keeps on typing my history. I suspect the doctor does not know what I look like, because he just keeps looking at the computer screen. Then I am asked to leave the [consultation] room and get the medicine. I just feel like a product in the production line of this medical factory. The doctors are only concerned about finishing their tasks in a short time, and I will be passed to the next step in this production line. I do not feel I am respected as a human in the follow-up, and they never pay much attention to what I say. [P14]

#### Feelings of not being understood

Often, the gender difference between the participants and their urologists made the participants feel that their urologists did not understand the extent of their suffering. More than half of the participants indicated that their urologists often adopted a male’s viewpoint when the participants expressed suffering. This made the participants’ communication experiences unpleasant. One of the participants recalled:

Many doctors cannot understand my difficulties. They just cannot understand how the disease has changed my life. They cannot make sense about how my frequency of going to the toilet can seriously affect my office work and home duties. Some doctors have even suggested that I consider focussing on home duties only because it is not a big deal for a married woman. They also commented that I should consider staying at home so that I can avoid being bothered by the symptoms. It is the 21st century now, but the doctors still believe a woman should stay at home and depend on her husband. I find it difficult to communicate with these doctors; they cannot understand the needs of female patients. [P21]

In some cases, the treatments suggested by the urologists contradicted the concerns and gender-related roles and responsibilities of the participants as women. The participants indicated that their urologists frequently failed to understand their social roles as women, as indicated by the following comment shared by one of the participants:

The doctors have constantly suggested that I try bladder instillation therapy. However, apart from the expensive treatment fees, I have to spend a whole day in the hospital for a series of treatments. This is really difficult for me as a working mother: I have a four-year-old daughter to care for. Also, I work a part-time job to help cover family expenses. However, the doctors cannot understand my position even though I have mentioned my concerns. They cannot understand why I have so many considerations even though there is a better treatment. Women have more family considerations before accepting treatment; they are men, so they may not be able to understand my concerns. [P18]

#### Perceived lack of empathy

The gender difference between the participants and their urologists led them to have contrasting views regarding the participants’ suffering. As women, the participants encountered many gender-related difficulties while undergoing treatment. However, these difficulties were not understood by their urologists. More than half of the participants reported that their urologists’ responses indicated a lack of empathy, as the following participant expressed:

The doctors do not care much about my bladder problem. They just think that my problems are normal for a woman of my age. One doctor even told me that it is a normal ageing process for a woman; he said if I can accept my problems as a normal ageing process, I will feel much better emotionally. He said because my problem is due to the ageing process, there is not much treatment he can do. Although I appreciate his honesty, I feel he is quite “cold-blooded” [a colloquial expression in Cantonese that means “apathetic”] towards my suffering. [P20]

In some cases, the urologists blamed the participants for their suffering and their perceived lack of improvement. The participants, because of their female gender roles, failed to follow the treatment regimen prescribed by their urologists. The participants were blamed by their urologists. However, the participants perceived the blame from the urologists to be a lack of empathy towards them, as indicated by the following recollection by one of the participants:

It is not easy for me to follow strictly the treatment plan, because I have my commitment to my family, children, and work. I can take the medicine accordingly, because it does not conflict with my schedule, but I really find it difficult to keep on receiving instillation and bladder training sessions. I am a working mother; therefore I have to put my family and my children at the first priority. If the treatment plan interferes with my responsibilities and my schedule, the only option for me is to give up. I understand that this will affect the efficacy [of my treatment], so I do not complain about the poor outcome at all; but the doctors blame me for treatment disobedience. They blame me for having so many excuses not to accept their treatment plans, and they blame me for lacking incentive to improve my condition. They just cannot understand a working mother’s concerns and difficulties. [P26]

#### Feelings of not being given autonomy to choose treatment approaches

More than half of the participants reported that they were not given autonomy in selecting a suitable treatment approach for themselves. One of the reasons was due to the perceived expert power of doctors and the perceived difference in hierarchy between patients and doctors. One of the participants shared the following perspective, which was common among the participants:

Different treatments can have different influences on my life, so of course I want to have some autonomy to choose the treatment that suits me most. However, I am just a patient, and doctors will think that I know nothing about treatment. It does not occur to them to allow me to choose the treatment that I prefer most. Sometimes I feel that doctors are afraid of patients who have a high level of education. They may feel that these patients are more troublesome and have more queries and requests to challenge them. In their mind, patients with a low level of education are good patients because they rarely ask questions and are more obedient. Therefore, I never let my doctors know about my education level. I do not want them to label me as a troublesome patient. [P23]

Another reason was due to the perceived gender difference between the participants and their urologists. Under the influence of the traditional patriarchal values, the participants, as women, did not feel comfortable with expressing their thoughts to male urologists. They tended to accept what their urologists had selected for them, because many of them were not accustomed to resisting decisions made by men, as indicated by the following comment by one of the participants:

Usually the doctors determine my treatment—I do not have a say—and I think the doctors do not expect that I would want to choose treatment approaches for myself, too. Also, I am a woman, and my doctors are men, so I think it is quite normal for a woman to listen to a man’s advice, just like I listen to my husband’s advice at home. I tend not to bargain too much with men. Therefore, I mostly accept what the doctors have assigned to me, although I am not happy with their suggestions and attitudes sometimes; after all, they are doctors, and they are men, so they will not listen to what a woman says. [P8]

## Discussion

Overseas studies have noted that among patients with OAB, women experience a lower quality of life than men do [11], and they are often more victimised and have worse illness experiences [31]. In the present study, the participants, all of whom were women, also suffered from unpleasant treatment experiences, particularly because of the gendered difficulties experienced while communicating with their urologists. The gender difference between the participants and their urologists led to large communication gaps between them, making the participants’ treatment-seeking experiences unpleasant. Embarrassment, feelings of not being treated seriously, not being understood, and not being given the autonomy to choose treatment approaches prevailed among the participants. Furthermore, the participants commonly perceived their urologists to lack empathy towards their suffering.

Embarrassment was common among the participants while communicating with their urologists. As noted in the literature, embarrassment is common among OAB patients [18, 32]. As demonstrated by the participants in the present study, the gender difference between them and their urologists frequently led them to feel embarrassed when they mentioned their symptoms during treatment. Because the participants preferred consulting a female doctor—even for diseases such as the common cold and influenza—the participants would presumably experience more emotional difficulty when communicating with a male urologist about their chronic bladder conditions, which were frequently perceived to be private and sensitive. Female patients in non-Western societies have a stronger preference for consulting female doctors for problems perceived to be sensitive [23, 33]. Moreover, because of the participants’ folk taxonomy [34] of diseases, urinary organs were often perceived to be closely related to the sexual and reproductive organs because of their biological location. Such folk taxonomies thus contributed to the participants’ sense of embarrassment when communicating with their male urologists about their bladder conditions.

Feeling embarrassed can deter a patient from seeking treatment [17]. As accounts in the present study show, the participants’ embarrassment hindered them from being honest and communicating effectively about their symptoms while undergoing treatment. Some participants entirely avoided mentioning the perceived embarrassing symptoms to their urologists. Such selective symptom reporting could mislead urologists in making accurate diagnoses as well as designing and adjusting treatment plans appropriately, which would have a marked impact on the treatment outcome. Although training more female urologists to address the special psychological and emotional needs of female patients may be a feasible solution, ensuring that all female patients receive chronic care from female urologists is unrealistic. Because of the lack of female urologists in Hong Kong, assisting patients with OAB in overcoming their sense of embarrassment is thus a key implication in OAB patient care.

A society’s social and cultural norms and values can influence doctor-patient communication. As demonstrated by the participants in the present study, the patriarchal values of Hong Kong society were reflected by how they communicated with their urologists. The professional roles of doctors are not gender-neutral, because the professional socialisation of doctors continues to perpetuate differences in gender roles [35]. The literature shows that male doctors tend to spend more time on technical practice behaviours, such as medical history taking and physical examination, rather than on psychosocial counselling, which is more frequently undertaken by female doctors [1]. Moreover, the communication style of male doctors is less patient-oriented [35]. The present study corresponds with these findings regarding how the gender role of male urologists and their gendered communication style often hindered them from being empathetic about the participants’ suffering, difficulties, treatment concerns, and social roles as women. As experienced by the participants, adopting men’s gaze during the consultation and treatment processes was not uncommon among their urologists. In many cases, the urologists advised the participants to remain at home when their condition or treatment interfered with their social roles.

Focussing on the domestic role is a traditional Chinese cultural value and expectation on women. Although many participants were in paid employment, they were also expected to manage domestic duties. As men, the urologists may have subconsciously followed this embedded traditional Chinese cultural expectation when prescribing treatment advice to the participants, rather than assisting them in maintaining their “double burden” social role. Doctors’ perceptions on the appropriate treatment for patients can be influenced by social ideologies and values [36, 37]. In a patriarchal society such as Hong Kong, where the position of women is often subordinate and disadvantaged [38], the participants’ feelings and expectations were not respected by their male urologists in many cases. Health care in Hong Kong is traditionally male-dominated. In certain specialties, such as urology, most doctors are men [6]. The position of the participants as female patients was therefore further subordinated under this power structure, making them feel demotivated about discussing their concerns with their urologists. The male-dominant urology field in Hong Kong had an impact on the participants’ activeness in their treatment-seeking behaviour. Although the participants looked forward to having more autonomy in their treatment, as women, some participants believed that it was normal for them to listen to and obey their urologists’ treatment decisions because they were men. Medical patriarchy was thus evident, following the embedded ideology of Hong Kong society.

In addition to the gender differential, the participants’ communication experiences with their urologists also intertwined with the power differential between doctors and patients in Hong Kong. As patients, the participants were rarely given the autonomy to select the treatment approaches for themselves, and their urologists usually held absolute power in this determination. Although some participants were allowed to make treatment decisions, they were questioned and blamed if their choices contradicted what their urologists perceived to be the best option. Downward communication from the urologists to the patients was the dominant communication mode experienced by the participants, and the participants dared not discuss or bargain with their doctors in deferring to the doctor-patient power differential. To avoid violating this social norm of the doctor-patient power differential, some participants intentionally concealed their background when communicating with their urologists if they believed that it potentially challenged this social norm.

Under such differentials in gender, power, and social hierarchy, the participants encountered considerable difficulties and unpleasantness when communicating with their urologists. The participants’ suffering was not understood, and was even underestimated and trivialised by their urologists. In many cases, the urologists tended to rationalise the participants’ condition as being “normal for middle-aged women.” Thus, conflict between the participants’ and their urologists’ understanding of the patients’ suffering was common. As Kleinman noted [39], a persistent gap exists between doctors’ and patients’ understanding of patient suffering. For doctors, their medical and biological training leads them to focus on their patients’ physical suffering or symptoms [39]. However, suffering has a broader meaning to patients. In addition to physical suffering, social suffering and psychological suffering are other critical concerns for patients [39]. Because of such perception gaps in their understanding of suffering, the participants experienced further frustration when communicating with their urologists.

The brief consultations experienced by the participants during treatment also played a critical role in affecting the quality of communication with their urologists. The short consultation time did not allow either the participants or their urologists to have sufficient time to communicate, which not only made it difficult for the urologists to understand the participants’ suffering but also led to the participants finding it difficult to understand the treatment. The literature shows that brief consultations typically result in low patient satisfaction with the treatment process [40] as well as a dehumanising communication experience and treatment environment [41]. This can endanger patients’ compliance and treatment outcome as a result.

The unpleasant communication experiences of the participants, however, were mostly caused by the social structure of gender, power, and social hierarchy differentials with their urologists, which could be difficult to alter. To improve the communication experiences of these patients, complementary support from nurse-led clinics and patient self-help groups can assist in filling the communication gap by providing a more humanised chronic care environment for these patients [42]. Moreover, because of the sensitivity and potential embarrassment resulting from the symptoms and examination procedures that patients with OAB are subjected to, urologists in Hong Kong are advised to be more aware of communication problems that might be caused by gender-specific issues when communicating with and performing examinations on patients of the opposite sex. Raising urologists’ awareness of gender-sensitive issues in communication could improve the treatment experiences of female patients suffering from OAB, which can benefit their treatment compliance and satisfaction.

### Limitations

The findings of this study were based on a sample of 30 women with OAB, who were recruited from a patient self-help group and had unpleasant experiences in communicating with their urologists. Therefore, further research on a larger sample of patients with OAB from other therapeutic settings might provide a more holistic understanding on this issue. Furthermore, whether male OAB patients have similar gendered communication difficulties during treatment processes remains unknown. Therefore, future research on male patients with female urologists may elucidate this problem from another perspective. In addition, this research was conducted by a single researcher, thus rendering the cross-checking of coding with other researchers impossible. To overcome such coding limitations, a recoding process was performed 1 month after the first coding.

## Conclusions

This study investigated the experiences of patients with OAB communicating with their male urologists during treatment. The gender and power differentials between the participants and their urologists made their communication unpleasant and difficult. Embarrassment, feelings of not being treated seriously, not being understood, and not being given autonomy in selecting treatment approaches prevailed among the participants. Furthermore, the participants commonly perceived their urologists to lack empathy towards their suffering. Such doctor-patient gender and power differentials were mostly contributed by the social and cultural values of patriarchy and the dominant position of doctors in Hong Kong. Encountering such medical patriarchy, the participants tended to accept this downward mode of communication from doctors to patients, despite the unpleasantness of such communication. Ineffective doctor-patient communication can endanger patients’ treatment compliance and thus negatively affect their treatment outcome. Although altering such social and cultural values would be different, complementary chronic care services provided by nurse-led clinics and patient self-help groups may be a feasible solution for improving the treatment experiences and thus the treatment outcomes of such patients.
